# Epicardial fat volume, an independent risk factor for major adverse cardiovascular events, had an incremental prognostic value to myocardial perfusion imaging in Chinese populations with suspected or known coronary artery disease with a normal left ventricular ejection fraction

**DOI:** 10.3389/fcvm.2023.1261215

**Published:** 2023-10-02

**Authors:** Le Yang, Wenji Yu, Peng Wan, JingWen Wang, Xiaoliang Shao, Feifei Zhang, Xiaoyu Yang, Yongjun Chen, Qi Li, Dan Jiang, Yufeng Wang, Qi Jiang, Jianfeng Wang, Yuetao Wang

**Affiliations:** ^1^ Department of Nuclear Medicine, The Third Affiliated Hospital of Soochow University, Changzhou, China; ^2^ Institute of Clinical Translation of Nuclear Medicine and Molecular Imaging, Soochow University, Changzhou, China; ^3^ Department of Nuclear Medicine, The first afflicted hospital of Ningbo University, Ningbo, China; ^4^ Department of Cardiology, The Third Affiliated Hospital of Soochow University, Changzhou, China

**Keywords:** EFV, coronary artery calcium, non-contrast CT, myocardial perfusion imaging, major adverse cardiovascular events (MACEs)

## Abstract

**Background:**

Most coronary artery disease (CAD) patients with a normal left ventricular ejection fraction (LVEF) experience a poor prognosis. Single-photon emission computerized tomography (SPECT)–myocardial perfusion imaging (MPI), a routine examination, is useful in assessing risk and predicting major adverse cardiovascular events (MACEs) in populations with suspected or known CAD. SPECT/CT is a “one-stop shop” examination, which, through non-contrast CT, can produce attenuation correction for MPI and obtain information on coronary artery calcium (CAC) and epicardial fat volume (EFV) simultaneously. This study aims to investigate the predictive and incremental value of EFV to MPI for MACE in Chinese populations with suspected or known CAD with a normal LVEF.

**Methods and results:**

We retrospectively studied 290 suspected or known CAD inpatients with a normal LVEF who underwent SPECT/CT between February 2014 and December 2017. Abnormal MPI was defined as a summed stress score ≥4 or summed difference score ≥2. EFV and CAC were calculated using non-contrast CT. The end date of follow-ups was in February 2022. The follow-up information was obtained from the clinical case notes of the patients or reviews of telephone calls. MACE was defined as cardiac death, late coronary revascularization ≥3 months after MPI, non-fatal myocardial infarction, angina-related rehospitalization, heart failure, and stroke. During the 76-month follow-up, the event rate was 32.0% (93/290). Univariate and multivariate Cox regression analyses concluded that high EFV (>108.3 cm^3^) [hazard ratio (HR): 3.3, 95% CI: 2.1–5.2, *P *< 0.000] and abnormal MPI (HR: 1.8, 95% CI: 1.1–2.8, *P = *0.010) were independent risk factors for MACE. The event-free survival of patients with high EFV was significantly lower than that of the low EFV group (log-rank test *P *< 0.001). In the subgroup with normal MPI, high EFV was associated with reduced event-free survival (log-rank *P *< 0.01), with a higher annualized event rate (8.3% vs. 1.9%). Adding high EFV to MPI could predict MACEs more effectively, with a higher concordance index (0.56–0.69, *P *< 0.01), higher global chi square (7.2–41.4, *P *< 0.01), positive integrated discrimination improvement (0.10, *P *< 0.01), and net reclassification index (0.37, *P *< 0.01).

**Conclusions:**

In Chinese populations with suspected or known CAD with normal LVEF, high EFV was an independent risk factor for MACE after adjusting for traditional risk factors, CAC and MPI. In subgroups with normal MPI, EFV could also improve risk stratification. Adding EFV to MPI had an incremental value for predicting MACE.

## Introduction

Coronary artery disease (CAD), the most common cause of death worldwide, has affected over 11 million patients in China. Left ventricular ejection fraction (LVEF) is a common parameter in assessing left ventricular global contraction function in clinical practice. The prognostic importance of LVEF has been demonstrated, and patients with low LVEF have increased mortality rates compared with those with normal LVEF (1). However, 314 patients died in a cohort study of 3,816 CAD patients with normal LVEF, and the risk stratification of CAD patients with normal LVEF has been poorly studied (2). Previous studies showed that single-photon emission computerized tomography (SPECT)-myocardial perfusion imaging (MPI) can effectively predict major adverse cardiovascular events (MACEs) by evaluating myocardial ischemia (3). However, SPECT-MPI has several disadvantages and a high false negative rate (4, 5). Even if the results of the MPI are normal, there is still a potential risk of cardiac death, myocardial infarction (MI), or late coronary revascularization (6, 7). Therefore, improving the risk stratification of populations with suspected or known CAD with normal LVEF and the prediction of MPI for MACE is necessary. As a “one-stop shop” examination, SPECT/CT can not only perform MPI but also improve the diagnosis of MPI by attenuation correction through non-contrast CT. In addition, it quantitatively assesses the coronary artery calcium score (CACS) and epicardial fat volume (EFV) simultaneously. CACS has been reported to be associated with MACE, which is controversial (8), and there are racial differences in CACS, with the lowest levels found in Asians (9).

Epicardial adipose tissue (EAT), which can be quantitatively measured using EFV, is a localized fat depot between the serous epicardium and pericardial fibers with paracrine and autocrine functions (10–13). By secreting various inflammatory factors, EAT can promote the occurrence and development of CAD, which may be an important predictor for MACE (14). Our previous study (15) demonstrated that the EFV was significantly associated with obstructive CAD in the Chinese population. In addition, a large population study (16) which included 5,743 Chinese patients showed that EFV improved the prediction of CAD, compared with clinical risk factors and CACS. There were significant racial differences in EFV (17, 18), and it was reported that EFV had an increased value in predicting MACE in western populations (8). The relationship between EFV and MACE and whether EFV can provide an incremental prognostic value to MPI in Chinese populations with suspected or known CAD with normal LVEF is still unknown.

This study aims to investigate the relationship between EFV and MACE and determine whether EFV could provide an incremental value above SPECT-MPI in predicting MACE in Chinese populations with suspected or known CAD with normal LVEF.

## Method

### Study population

**Figure 1:**
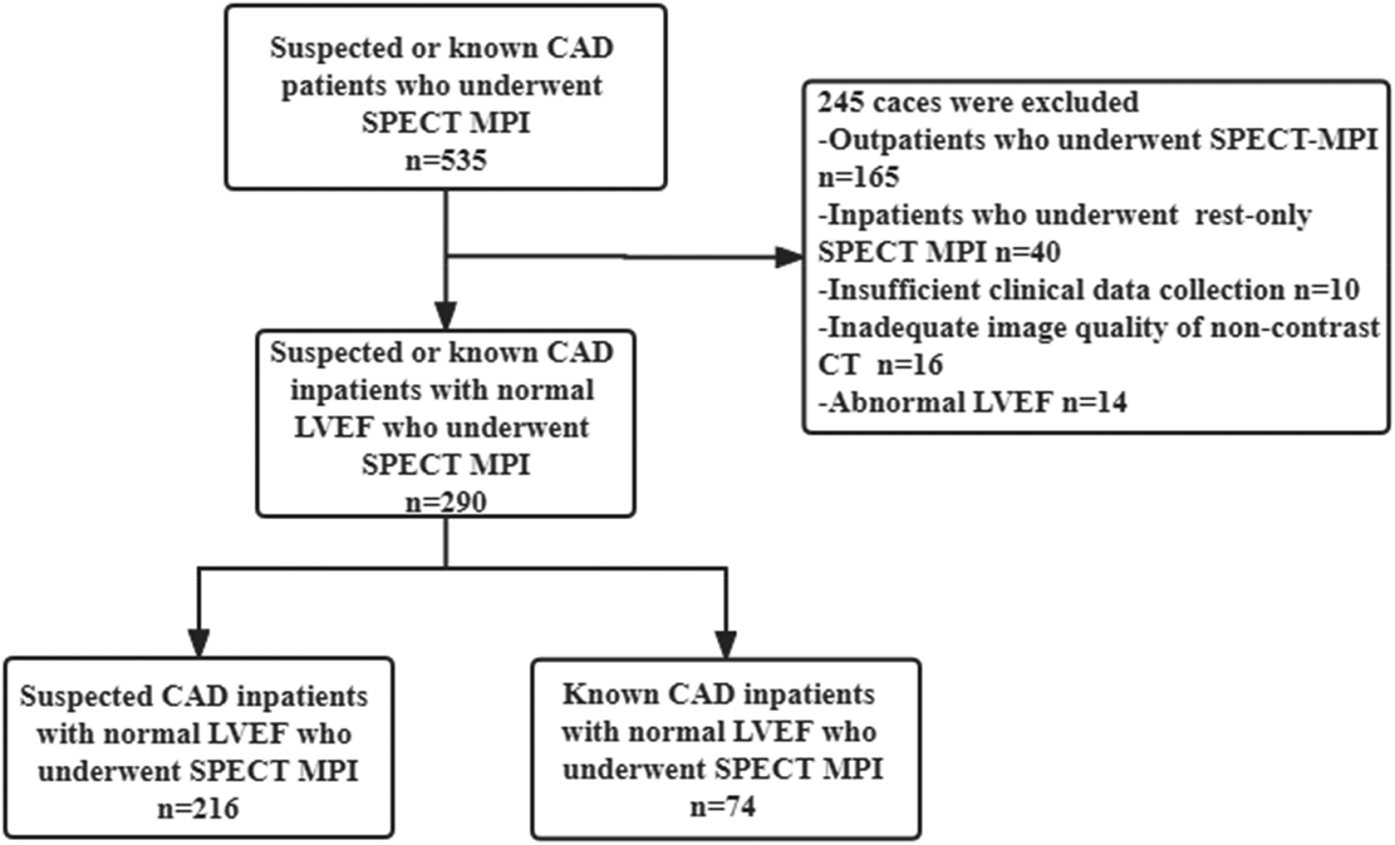
Flow chart of the population selection in this study.

The study was retrospective, and we consecutively enrolled 535 suspected and known CAD patients who underwent SPECT/CT to evaluate myocardial ischemia between February 2014 and December 2017 at the Third Affiliated Hospital of Soochow University. SPECT/CT is a “one-stop shop” examination, which can not only perform MPI but also produce attenuation correction and acquire CAC and EFV simultaneously through a non-contrast CT scan. Initially, 165 outpatients were excluded, followed by 40 inpatients that underwent rest-only SPECT-MPI. A total of 10 patients had incomplete clinical data or information, and 16 patients had inadequate image quality of non-contrast CT scans. Finally, the analysis included 290 inpatients. Figure 1 shows the flow chart. Patients with a history of MI, percutaneous coronary intervention (PCI), or coronary artery bypass grafting (CABG) were defined as known CAD (19).

### Clinical data

Body mass index (BMI) was calculated as weight (kg) divided by height squared (m^2^). Hypertension was defined as systolic blood pressure ≥140 mmHg, diastolic blood pressure ≥90 mmHg, or use of antihypertensive medication. Hypertension was defined as having a history of hypertension or receiving antihypertensive therapy. Diabetes mellitus was defined by random blood glucose ≥11.1 mmol/L or fasting blood glucose ≥7 mmol/L or 2-h postprandial blood glucose (oral glucose tolerance test) ≥11.1 mmol/L or HbA1c ≥6.5%. Data for LVEF were obtained by echocardiography before SPECT-CT. LVEF ≥ 50% was defined as normal; LVEF<50% was defined as abnormal. Hyperlipidemia was defined as a history of hyperlipidemia, total cholesterol >5.2 mmol/L, low-density lipoprotein cholesterol >3.36 mmol/L, or use of lipid-lowering medications. Current smokers were defined as smoking one cigarette per day for at least half a year.

### SPECT/CT imaging acquisition

SPECT-MPI was performed according to indication and a 2-day standard imaging protocol. Medications such as nitrates and beta-blockers were not allowed before testing. Approximately 69.6% of the patients underwent stress testing with exercise stress MPI based on the Bruce protocol, and 30.4% received pharmacological stress MPI with adenosine intravenously (i.v.) infused at 0.14 mg/kg/min for 6 min. Any products containing methylxanthines, including caffeinated coffee, tea, or other caffeinated beverages, caffeinated medicines, and theophylline, were avoided before using adenosine intravenously for at least 12 h. MPI acquisitions were initiated 60–90 min after intravenous injection of 99mTc-MIBI (radiochemical purity 95%, injected dose of 740–925 MBq). MPI was performed using a dual-head camera at a 90° angle (SPECT/CT Symbia T16, Siemens Medical Systems, Erlangen, Germany). This setup included a high-resolution low-energy parallel hole collimator and a 180° orbit (from the right anterior oblique of 45° to the left posterior oblique of 45°). MPI was produced using a matrix of 128 × 128, magnification of 1.45, and a 20% window centered on the 140 keV peak energy. We used the filtered back projection method (Butterworth filter with cutoff frequency of 0.35 and order of 5) to reconstruct MPI and acquire horizontal long axis, vertical long axis, and short axis images. A non-contrast CT chest scan was acquired for attenuation correction after MPI. EFV and CACS were obtained through the non-contrast chest CT scan after rest MPI. The following scan parameters were then executed: 60%–80% RR, tube voltage: 130 kV, tube current: 100 mAs, and thickness: 3 mm. Each scan extended from the plane underneath the tracheal carina to the place 1–2 cm below the heart diaphragmatic surface, which is almost 20 cm.

### Imaging data processing and analysis

Two experienced nuclear medicine physicians, unaware of the clinical information of the patients, independently performed the imaging. In cases where the two nuclear medicine physicians had differing opinions, a third expert was consulted to establish consensus results. Myocardial perfusion images were divided into 17 segments, and a 5-point continuous scoring system was used (0: normal, 1: mild abnormality, 2: moderate abnormality, 3: severe abnormality, and 4: segmental loss of uptake). The summed stress score (SSS) and summed rest score (SRS) were calculated from all abnormal segments. The summed difference score (SDS) was determined by subtracting SRS from SSS. Abnormal MPI was defined as SSS ≥4 or SDS ≥2 (20).

### CAC

CAC was identified as the dense area in the coronary arteries that exceeded the 130 HU threshold measured using Agatston automated analysis software. For patients who previously underwent coronary artery bypass surgery, the Agatston score was calculated using only the native vessel. Segments previously implanted with coronary stents were excluded from CAC calculations (21).

### EFV

EFV is identified as any adipose tissue located within the pericardial sac. The specific methods used are as follows: initially, the upper limit of the slice is determined by the bifurcation of the pulmonary artery, while the lower limit of the slice is defined as the last slice that contains any part of the heart. The pericardial contour was then manually traced using the reconstructed axial slices. The sum of all pixels within the −190 to −30 HU window of the region of interest was defined as a fat voxel. EFV was calculated by multiplying the sum of the cross-sectional areas of the fat by the slice thickness (22). The whole process was performed using software (Syngo, Siemens Medical Solutions). The intra-class correlation coefficient for EFV was 0.940 (95% CI: 0.855–0.976, *P* < 0.001).

### Radiation exposure

The standard effective radiation dose of 99mTc-MIBI imaging (including rest and stress imaging) was 9–12 mSv. The dose of the non-contrast CT scan (voltage, 130 kV; tube current, 100 mA) used to assess CACS and EFV was 1–2 mSv.

### Patient follow-up and MACE

The follow-up information was obtained from patient clinical case notes or telephone reviews. All endpoints were determined by consensus between two blind reviewers. The follow-up deadline was February 2022. MACE was defined as cardiac death, late coronary revascularization ≥3 months after MPI (23), non-fatal MI, angina-related rehospitalization, heart failure, and stroke. Cardiac death was defined as death due to acute MI, ventricular arrhythmia, or cardiogenic shock. Late coronary revascularization ≥3 months after SPECT-MPI included CABG and PCI. Those with early coronary revascularizations within 3 months from SPECT-MPI were excluded from the MACE since these procedures were likely to be triggered by the SPECT-MPI test result itself. Non-fatal MI was diagnosed based on symptoms, enzymes (creatine kinase levels, troponin T or I), and ECG signs (23). Angina-related rehospitalization was defined as chest pain or chest pain equivalent with dynamic ECG changes such as ST depression or T-wave inversion without abnormal cardiac biomarkers and characterized by rest symptoms, new-onset angina (less than 2 months), or an increased duration or severity of earlier stable angina symptoms. The diagnosis of heart failure was confirmed following the established criteria (24). Stroke was identified as an ischemic cerebral infarction induced by a thrombotic or embolic occlusion of any major intracranial artery (25).

### Statistical analysis

Continuous variables are expressed as mean ± standard deviation, and categorical variables are expressed as numbers and percentages. Comparisons were analyzed for significant differences using the *t*-test, Mann–Whitney *U*-test, or chi-squared test. Time-dependent receiver-operating characteristic (ROC) (survival ROC) curves were applied to assess the EFV prognostic power, and the optimal cutoff value of EFV for predicting MACE was 108.3 cm^3^, which was used to split the data into two categories. The Kaplan–Meier (K–M) method was used to analyze the event-free survival rate over time, and the differences between survival curves were compared using the log-rank test. The point-biserial correlation coefficient was used in correlation analysis. A collinearity analysis was performed to exclude all indicators with a variance inflation factor of >5. Cox proportional hazards regression models were used to identify predictors of MACE, and hazard ratios (HR) with 95% confidence intervals (CI) were calculated. Univariable and multivariable Cox regression models were created to assess the association between EFV and MACE. The global chi-squared test, C-index, net reclassification index (NRI), and integrated discrimination improvement (IDI) were used to analyze the incremental prognostic values of EFV to MPI. All statistical analyses were performed using a commercially available software package (R).

## Result

### Study population

This analysis included 290 inpatients (mean age: 67.62 ± 9.52 years, male: 57.9%). Table 1 summarizes their detailed characteristics. The average value of LVEF was 62.7%. The maximum value of EFV was 247.89 cm^3^, and the minimum value of EFV was 37.3 cm^3^. Mean ± standard deviation of EFV was 114.56 ± 36.63 cm^3^. The median (Q1, Q3) was 107.94 (88.69, 137.73) cm^3^. Abnormal MPI occurred in 77 (26.5%) patients, and MACEs occurred in 93 patients (32.0%), including seven cardiac deaths, 15 non-fatal MIs, 10 revascularizations, 48 angina-related rehospitalizations, two heart failures, and 11 strokes.

**Table 1:** Baseline characteristics of the study cohort (*n* = 290).

	Over all (*N* = 290)	MACE (*N* = 93)	No MACE (*N* = 197)	*P*-value
Age (years, mean + SD)	67.5 ± 9.5	69.5 ± 9.0	66.6 ± 9.7	0.018
Male gender, *n* (%)	168 (57.9%)	64 (68.8%)	104 (52.8%)	0.010
BMI (kg/m^2^, mean + SD)	25.4 ± 13.0	25.4 ± 2.7	25.4 ± 15.8	0.980
Hypertension, *n* (%)	182 (62.7%)	66 (71.0%)	116 (58.9%)	0.047
Diabetes mellitus, *n* (%)	105 (36.2%)	39 (41.7%)	66 (33.5%)	0.163
LVEF (%, mean + SD)	62.7 ± 5.0	62.1 ± 5.8	63.1 ± 4.7	0.115
Hyperlipidemia (%)	118 (40.6%)	37 (39.8%)	81 (41.1%)	0.829
Current smoker, *n* (%)	93 (32.0%)	35 (37.6%)	58 (29.4%)	0.163
EFV (cm^3^, mean + SD)	114.5 ± 36.5	132.4 ± 32.7	106.1 ± 3.3	<0.001
CAC, *n* (%)	130 (44.8%)	57 (61.3%)	73 (37.1%)	<0.001
CACS (cm^3^, mean + SD)	86.9 (0,32.2)	131.8 (0,25.6)	66.0 (0,22.4)	<0.001
Abnormal MPI, *n* (%)	77 (26.5%)	35 (37.6%)	42 (21.3%)	<0.001

BMI, body mass index; CACS, coronary artery calcium score; EFV, epicardial fat volume; LVEF, left ventricular ejection fraction; MPI, myocardial perfusion imaging; MACE, major adverse cardiovascular events.

### Differences between the MACE group and without MACE group

Among patients who experienced MACE, male proportion, hypertension, diabetes mellitus (DM), hyperlipidemia, current smoker, high EFV (EFV >108.3 cm^3^), CAC, and abnormal MPI were more common than in patients without MACE (*P* for all <0.05) (Table 1).

### The relationship between EFV and MACE

**Figure 2:**
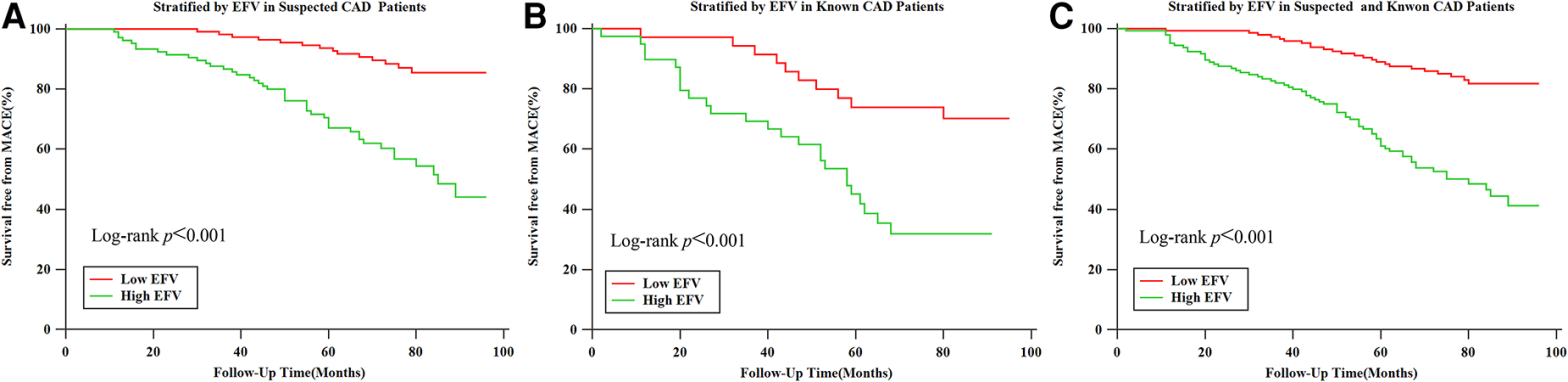
Kaplan–Meier survival curves for patients with low (≤108.3 cm^3^) and high (>108.3 cm^3^) EFV. (**A**) Kaplan–Meier survival curves for patients with low (≤108.3 cm^3^) and high (>108.3 cm^3^) EFV in suspected CAD group populations. (**B**) Kaplan–Meier survival curves for patients with low (≤108.3 cm^3^) and high (>108.3 cm^3^) EFV in known CAD populations. (**C**) Kaplan–Meier survival curves for patients with low (≤108.3 cm^3^) and high (>108.3 cm3) EFV in suspected and known CAD populations.

In the K–M survival curve analysis, the event-free survival rate of patients with high EFV was significantly lower than that of patients with low EFV, and there was a significant difference between the curves based on the log-rank test (*P *< 0.001) (Figure 2C). Subgroup analysis showed that patients with high EFV were more likely to experience MACE, and this trend was consistent in the suspected and known CAD group (both log-rank *P*-values <0.001, Figures 2A, B). In the univariate Cox proportional hazards regression analysis, age (HR: 1.0, 95% CI: 1.0–1.0), male proportion (HR: 1.8, 95% CI: 1.2–2.8), high EFV (HR: 3.1, 95% CI: 2.1–4.7), CAC (HR: 2.2, 95% CI: 1.5–3.4), and abnormal MPI (HR: 1.8, 95% CI: 1.2–2.7) were associated with MACE (*P* for all <0.05). In univariate and multivariate Cox proportional hazards regression analyses employing traditional risk factors (age, male gender, BMI, current smoking status, presence of hypertension, DM, and hyperlipidemia), CAC, high EFV, and abnormal MPI, only high EFV (HR: 3.3, 95% CI: 2.1–5.2, *P* < 0.001) and abnormal MPI (HR: 1.8, 95% CI: 1.1–2.8, *P* = 0.010) were significantly associated with MACE (Table 2). Patients with abnormal MPI exhibited lower event-free survival rates in the K–M survival curve compared with those with normal MPI, and these differences were significant based on the log-rank test (*P *< 0.001) (Supplementary Figure 1).

**Table 2:** Univariable and multivariable Cox proportional hazard regression analyses for predicting MACE.

	Univariate HR (95% CI)	*P*-value	Multivariate HR (95% CI)	*P*-value
Age (years, mean + SD)	1.0 (1.0–1.0)	0.025	1.0 (1.0–1.0)	0.722
Male gender	1.8 (1.2–2.8)	0.008	1.0 (0.7–2.0)	0.473
BMI	1.0 (1.0–1.0)	0.934	1.0 (1.0–1.0)	0.661
Hypertension	1.5 (1.0–2.4)	0.064	1.0 (0.6–1.7)	0.860
Diabetes mellitus	1.3 (0.9–2.0)	0.179	1.1 (0.7–1.8)	0.552
Hyperlipidemia	0.8 (0.5–1.3)	0.397	0.6 (0.4–1.0)	0.035
Current smoker	1.4 (0.9–2.2)	0.108	1.0 (0.6–1.6)	0.974
High EFV	3.1 (2.1–4.7)	<0.000	3.3 (2.1–5.2)	<0.000
CAC	2.2 (1.5–3.4)	<0.000	1.5 (1.0–2.5)	0.051
Abnormal MPI	1.8 (1.2–2.7)	0.007	1.8 (1.1–2.8)	0.010

BMI, body mass index; EFV, epicardial fat volume; CAC, coronary artery calcium; HR, hazard ratio; MPI, myocardial perfusion imaging; MACEs, major adverse cardiovascular events.

### Incremental predictive value of EFV to MPI for MACE

**Figure 3:**
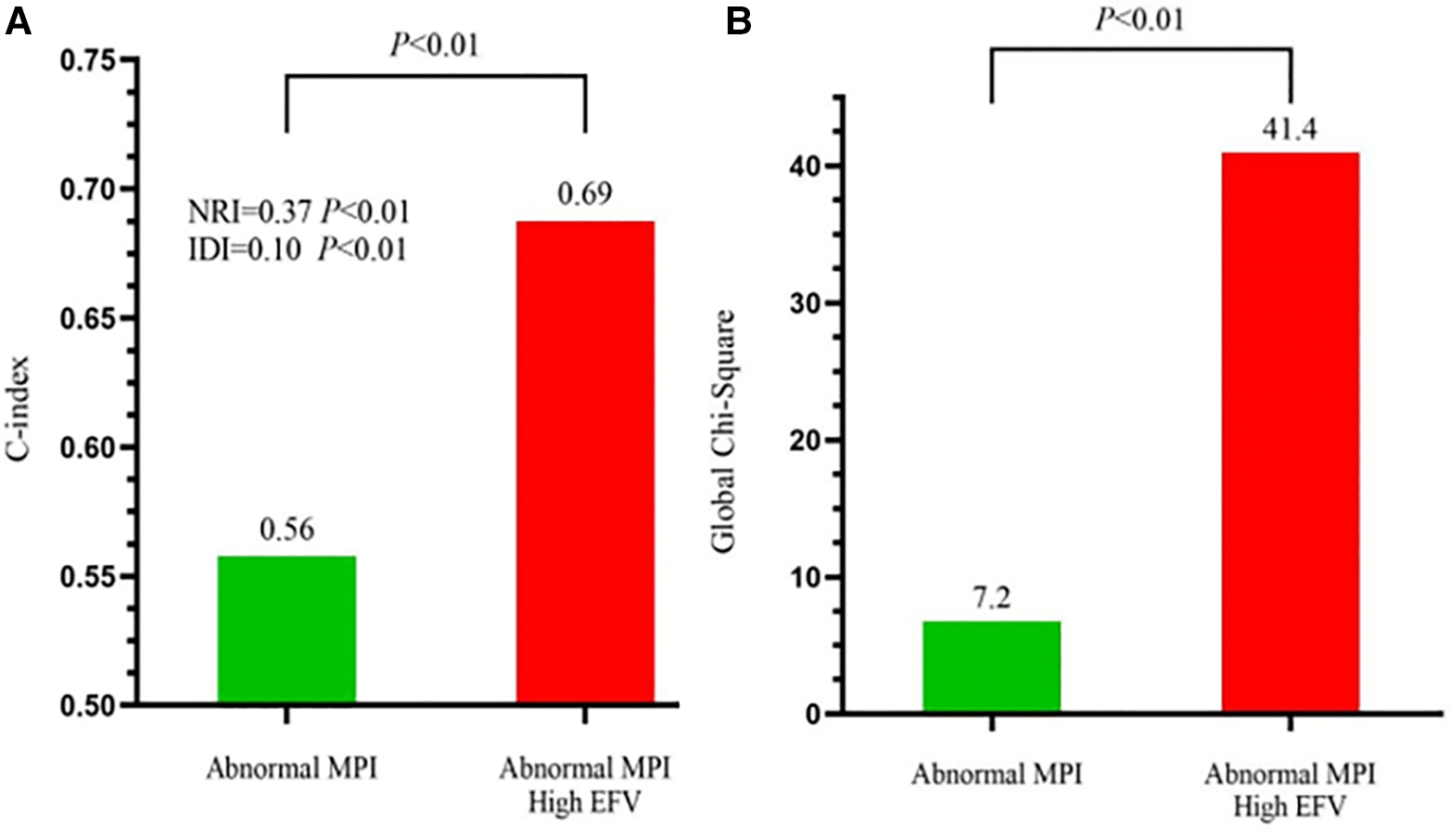
Incremental predictive value of EFV to MPI for MACE. (**A**) Receiver-operating characteristic curve analysis to evaluate the incremental prognostic value of EFV >108.3 cm^3^ over abnormal MPI. C-index, concordance index. (**B**) Global chi-squared test analysis to evaluate the incremental prognostic value of EFV >108.3 cm^3^ over abnormal MPI.

To further assess the predictive value of EFV as an adjunct to MPI for MACE, we calculated the global chi-squared score, concordance index, IDI, and NRI (Figure 3). It has shown that after adding EFV to MPI, the global chi-squared score increased significantly from 7.2 to 41.4 (*P *< 0.01), and the concordance index increased from 0.59 to 0.69 (*P *< 0.01). A positive IDI of 0.10 and positive NRI of 0.37 (both *P *< 0.01) were noted.

### Risk stratification analysis

**Figure 4:**
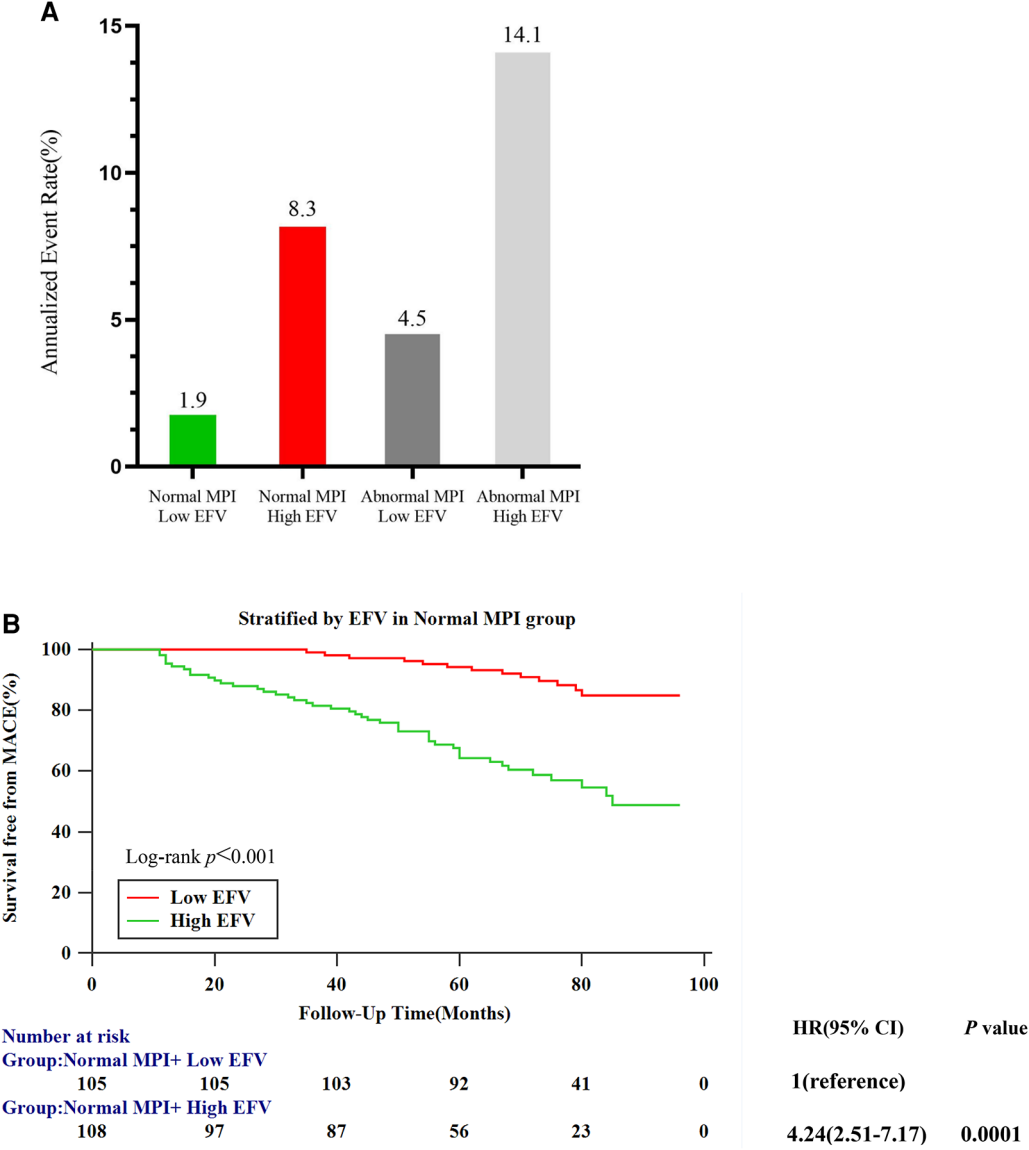
Risk stratification analysis. (**A**) Annualized event rates for MACEs by the combination of MPI and EFV. (**B**) Kaplan–Meier survival curves for patients with low (≤108.3 cm^3^) and high (>108.3 cm^3^) EFV in the normal MPI subgroup.

**Figure 5:**
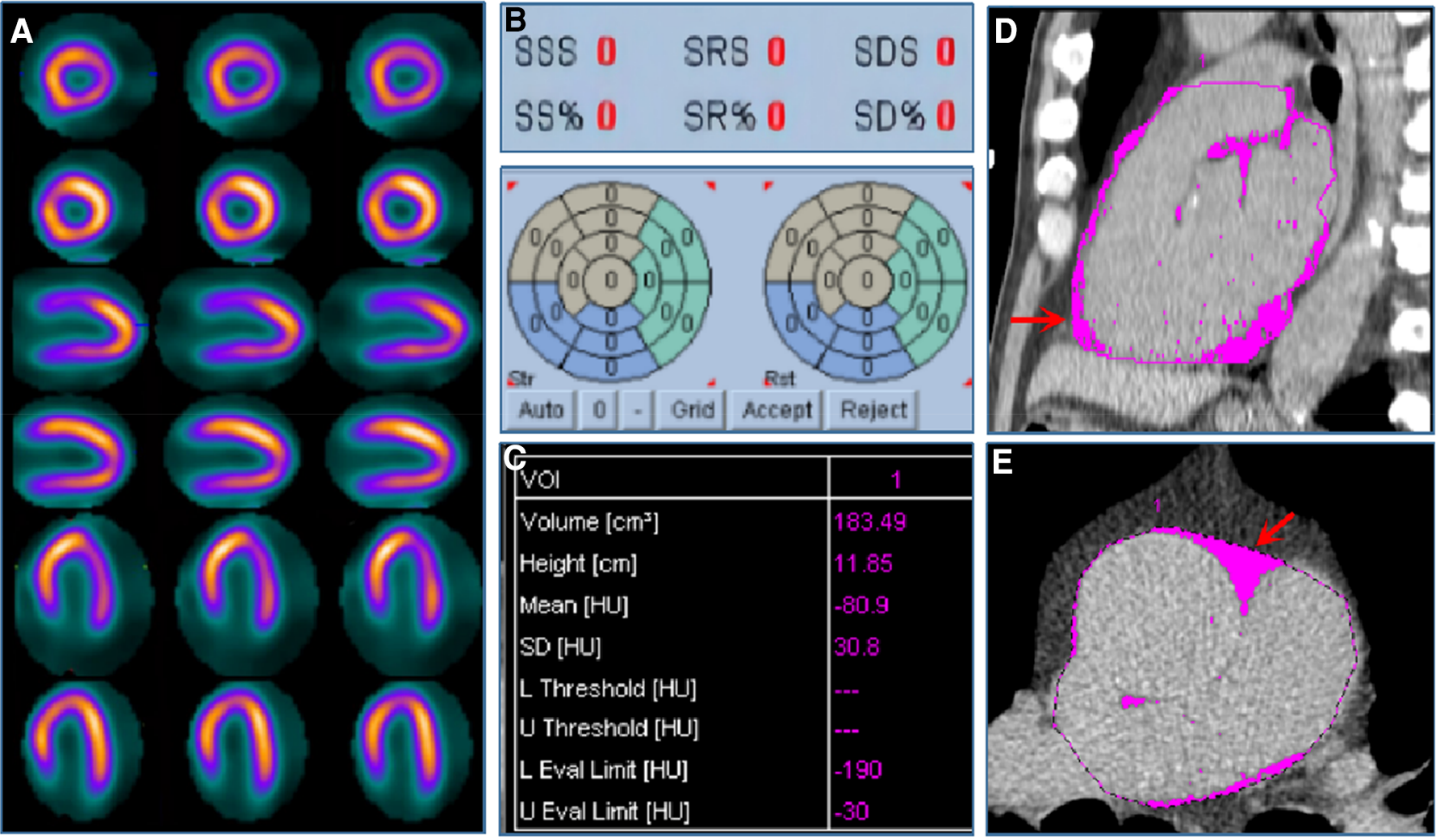
Case example. A 57-year-old male patient was admitted to the hospital with atypical chest pain for half a month. The patient was diagnosed with suspected CAD and received stress–rest MPI. (**A**) shows stress (the odd row) and rest (the even row) MPI of the short axis, vertical long axis, and horizontal long axis images, respectively, indicating the normal MPI with SDS equaled to 0 (**B**). (**C**) shows EFV was 183.49 cm^3^. (**D,E**) show EAT in pink of coronal and sagittal views from non-contrast CT scan.

Figure 4A shows the annualized event rates among four subgroups stratified by MPI (normal and abnormal) and EAT volume (cutoff value: 108.3 cm^3^), which were 1.9%, 8.3%, 4.5%, and 14.1% in normal MPI and low EFV, normal MPI and high EFV, abnormal MPI and low EFV, and abnormal MPI and high EFV, respectively. In the normal MPI subgroup, K–M survival curves reveal a higher cumulative event rate for patients with high EFV (log-rank *P* < 0.001). High EFV was associated with a higher HR for MACE (HR: 4.24, 95% CI: 2.51–7.17, *P* = 0.001) (Figure 4B). Figure 5 is the case example.

## Discussion

In this study, we examined the association of EFV with MACE in a Chinese population with suspected and known CAD with normal LVEF. K–M curve analysis based on the log-rank test indicated that patients with high EFV have a higher risk of MACE. Through Cox univariate and multivariate regression analyses, we observed that high EFV was an independent risk factor for MACE after adjusting for traditional risk factors, CAC, and abnormal MPI. Adding EFV to MPI had a significantly incremental value for predicting MACE. Among patients with normal MPI, the high EFV group had a higher risk of MACE.

CAD is the major cause of mortality in the Chinese population and is a serious threat to the national health. It is well known that patients with reduced LVEF have a poor prognosis. However, in the early compensatory period, LVEF is usually normal, even though myocardial impairment or ischemia may already exist (26). A study has shown that 8.2% of patients also experienced death in CAD patients with normal LVEF (2), and the risk stratification of CAD patients with normal LVEF has been poorly studied. Effective risk stratification of suspected or known CAD patients with normal LVEF is important in improving the prognosis. As a classic examination, MPI is a commonly used imaging tool for the non-invasive diagnosis of myocardial ischemia and risk stratification. In the present study, we concluded that abnormal MPI was significantly associated with MACE after performing Cox regression univariate and multivariate analyses. However, MPI has low spatial resolution, and balanced ischemia can lead to false negatives, which may affect risk stratification and clinical decision-making. Previous studies found that CAC, obtained from the one-stop shop SPECT/CT, was an independent predictor of MACE, and it can supply incremental value to MPI for predicting events (8). In this study, CAC was significantly associated with MACE in univariate analysis. However, statistical significance was lost after performing a multivariate analysis adjusting for clinical risk factors, possibly due to the racial differences and patients enrolled. A multiethnic study of atherosclerosis showed significant racial differences in CACS, and the Chinese population had the lowest CACS for both men and women (9). Similar to our result, after adjusting for traditional risk factors, CAC could not effectively predict MACE in Asian participants (27).

EAT, an ectopic adipose tissue directly surrounding the coronary arteries, can secrete various cytokines and adipokines through paracrine and endocrine pathways, damaging the myocardium and coronary arteries (28). EAT is not evenly distributed around the heart. It is most commonly found around the right ventricle, the right atrioventricular groove, and the apex of the heart. In contrast, it is least commonly found around the left anterior descending artery and the left atrioventricular groove. EAT can be quantitatively evaluated by EFV, which can be measured from the same CT image used to measure CACS. This approach has been given more and more attention recently. Previous studies have shown that elevated EFV may be associated with early atherosclerosis, and patients with abnormal EFV may be in a subclinical atherosclerotic state (29). In addition, EFV is important in affecting vascular function, especially in response to plaque changes and in influencing microvessels (30). The association between EFV and MACE has not been elucidated yet. It was demonstrated that high EFV may be an independent predictor of future coronary events in patients without proven CAD (23, 31). In addition, Mahabadi et al. (32) concluded that EFV is associated with fatal and non-fatal coronary events in the general population independent of traditional cardiovascular risk factors and provided additional information above the CACS. On the contrary, Possner et al. (33) found that EFV was associated with MACE and may improve risk stratification beyond clinical cardiovascular risk factors in populations with suspected and known CAD. However, when CACS and/or SPECT-MPI results were available, EFV did not provide any additional clinically relevant prognostic value. It is worth noting that their study population consists of Caucasians, and they did not consider racial differences. The distribution of EFV has obvious racial and geographical differences (34, 35), and EFV was significantly higher in Asians compared with Caucasians. Previous large cohort studies have confirmed that EFV was associated with obstructive CAD in the Chinese population (16). In addition, our previous results support the notion that the accumulation of EAT plays a critical role in the development of CAD, but the prognostic value of EFV for Chinese patients with suspected and known CAD with normal LVEF has not been extensively studied. In the present study, we adopted 108.3 cm^3^ as the cutoff value of EFV based on the time-dependent ROC (survival ROC) curves. We demonstrated that patients with high EFV have a higher risk of MACE, and EFV was an independent prognostic factor of MACE in the Chinese population with suspected and known CAD with normal LVEF after adjusting for traditional risk factors, CAC, and abnormal MPI. Our results showed that among patients with normal MPI, those in the high EFV group had a higher risk of MACEs.

This study followed up suspected and known CAD subjects with normal LVEF for approximately 6 years and showed that adding EAT to MPI had a significantly incremental value for predicting MACE. To our knowledge, this is the first study to demonstrate that EFV can provide an incremental value for MPI in predicting the prognosis in the Chinese population with suspected and known CAD with normal LVEF. The most provocative finding is that the high EFV group has significantly worse long-term outcomes, even in a normal SPECT group. EFV represents a novel therapeutic target for the treatment of CAD. Statins have been shown to induce a reduction of EFV and a direct anti-inflammatory effect on EAT (36). High EFV leads to a poor prognosis in Chinese populations with suspected or known CAD with normal LVEF, which may further improve their risk stratification. Through treatment and with the reduction of EFV, it had the potential to improve outcomes, which warrants additional investigation.

## Limitation

Several limitations were found in this study. First, this study is based on a single-center retrospective study, and there may be bias in the included population. Second, this study did not measure the perivascular Fat Attenuation Index (FAI), and we will quantitatively measure the FAI in the future to evaluate whether FAI could add a prognostic value beyond that provided by EFV, MPI, and CACS. Third, we did not consider the prognostic impact of drug use such as statins and aspirin. Further prospective observational studies are needed to evaluate the effect of statins on improving prognosis by reducing EFV. Fourth, all subjects were of the Chinese population with suspected and known CAD with normal LVEF, and the findings cannot be extrapolated to other populations. Further investigations are required to clarify the validity of our findings in patients with reduced LVEF. In addition, only a few patients (*n* = 8) underwent reduced LVEF during the follow-up, which is insufficient for statistical analysis. Follow-up studies are warranted to clarify the relationship between changes in LVEF and primary endpoint further. Finally, we did not distinguish fat types such as brown fat and white fat. Inflammatory white adipose tissue and anti-inflammatory brown adipose tissue may have different effects on MACEs (37, 38).

## Conclusion

In Chinese populations with suspected or known CAD with normal LVEF, EFV was the independent risk factor for MACE after adjusting for traditional risk factors, CAC, and MPI. In subgroups with normal MPI, EFV could also improve risk stratification. Adding EFV to MPI had an incremental value for predicting MACE.

## Data Availability

The raw data supporting the conclusions of this article will be made available by the authors, without undue reservation.

## Supplementary material

Supplementary Figure 1 Kaplan–Meier survival curves by MPI for the prediction of MACEs in suspected and known CAD.
