# Supplementary material for: Epicardial fat volume, an independent risk factor for major adverse cardiovascular events, had an incremental prognostic value to myocardial perfusion imaging in Chinese populations with suspected or known coronary artery disease with a normal left ventricular ejection fraction
Source: Front Cardiovasc Med. 2023 Oct 2;10:1261215. doi: 10.3389/fcvm.2023.1261215 (PMC10577423; doi:10.3389/fcvm.2023.1261215)

Supplement 1 Kaplan-Meier survival curves by MPI for prediction of MACEs in suspected and known CAD

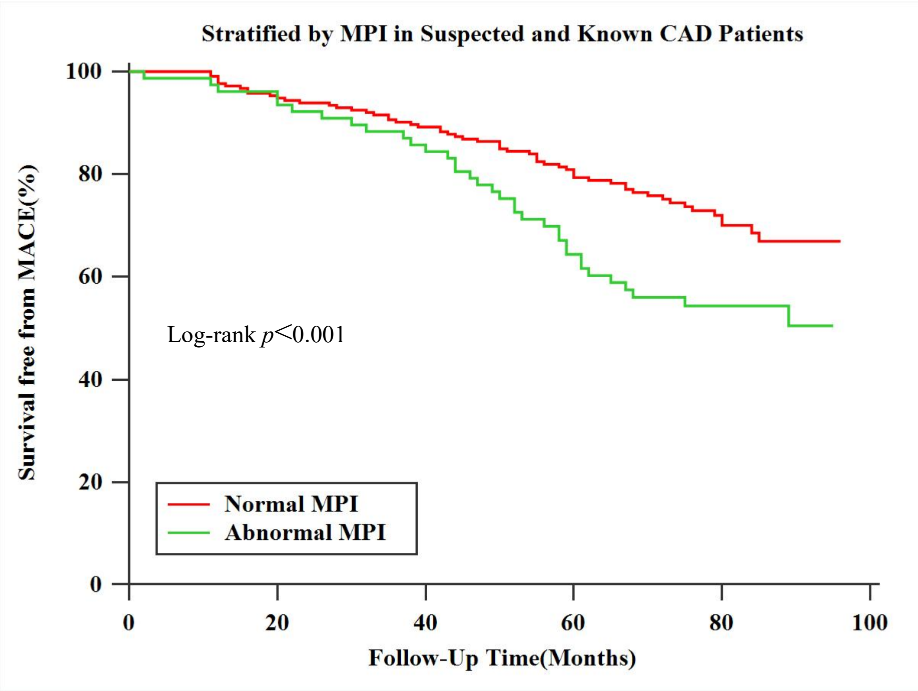

Supplement: Supplementary Figure 1 — Kaplan–Meier survival curves by MPI for the prediction of MACEs in suspected and known CAD. [file Image1.pdf]
